# Validation of the Medicare-Enhanced Laboratory and Demographics (MELD™) Dataset: A Comprehensive Psychometric, Epidemiologic, and Predictive-Utility Assessment of a 60-Million-Patient Real-World Evidence Resource

**DOI:** 10.36469/001c.162896

**Published:** 2026-06-11

**Authors:** Onur Baser

**Affiliations:** 1 Graduate School of Public Health City University of New York, New York, NY

**Keywords:** real-world evidence, Medicare, electronic medical records, data validation, psychometrics, record linkage, health economics and outcomes research

## Abstract

**Background:**

Real-world evidence studies increasingly rely on integrated data resources that combine Medicare fee-for-service claims with electronic medical records (EMRs), laboratory results, and patient-reported outcomes. The Medicare-Enhanced Laboratory and Demographics (MELD™) dataset, developed by Columbia Data Analytics, spans 2020 through 2024 and contains 60 million unique patients, roughly 1 billion visits, and more than 241 000 clinicians. Its data-quality properties must be characterized before regulatory-grade use.

**Objective:**

To provide a preregistered, multidomain validation of MELD, quantifying internal consistency, concurrent validity against Centers for Medicare & Medicaid Services (CMS) benchmarks, construct validity, record-linkage quality, missing-data mechanisms, temporal stability, and predictive validity across 20 indication cohorts.

**Methods:**

An analytic cohort of 32 118 604 patients with at least 12 months of continuous Medicare enrollment was derived. Internal consistency used Cronbach’s α and Kuder-Richardson KR-20. Concurrent validity compared MELD with CMS benchmarks using bias, mean absolute percentage error, Lin’s concordance correlation coefficient, and Bland-Altman limits of agreement. Construct validity used exploratory and confirmatory factor analysis. Record linkage used Fellegi-Sunter probabilistic matching. Predictive validity was assessed for 12-month mortality and 30-day readmission using the area under the receiver operating characteristic curve (AUROC), Brier score, and calibration slope/intercept.

**Results:**

Internal consistency was high (Cronbach’s α, 0.87; KR-20, 0.84). MELD replicated CMS prevalence with mean bias, −1.32 per 1000; Lin’s concordance correlation coefficient, 0.993 (95% confidence interval, 0.986-0.997); and mean absolute percentage error, 3.1%. The 3-factor confirmatory model achieved excellent fit (comparative fit index, 0.962; root mean square error of approximation, 0.041; standardized root mean square residual, 0.036). Fellegi-Sunter linkage produced a 94.7% match rate with false-match probability <0.3%. Weighted κ between International Classification of Diseases, Tenth Revision claims and EMR problem lists ranged 0.66 to 0.91. AUROC was 0.821 for mortality and 0.783 for readmission, with calibration slopes 0.97 and 0.94.

**Conclusions:**

MELD demonstrated strong psychometric, epidemiologic, and predictive properties, supporting use in health economics and outcomes research for Medicare-aged and adjacent populations. Limitations include residual missingness of race/ethnicity (26%) and EMR fragmentation outside networked provider groups.

## INTRODUCTION

Real-world data (RWD) have become foundational to health-economics and outcomes research (HEOR), pragmatic clinical trials, regulatory review, and value-based payment. The US Food and Drug Administration (FDA) 21st Century Cures Act and subsequent real-world evidence (RWE) framework require sponsors to characterize the relevance and reliability of the underlying data source before an RWE submission may be considered.^1,2^ Within the RWD landscape, administrative claims occupy a distinctive position. Unlike electronic medical records (EMRs), which capture encounters only at a specific provider or network, claims data are generated every time a covered service is billed to a payer, producing a near-complete longitudinal record of healthcare utilization, pharmacy fills, procedures, and costs for the enrolled population. Claims have well-defined population denominators (enrollment files), standardized coding systems (*International Classification of Diseases, Tenth Revision, Clinical Modification* [ICD-10-CM]; Current Procedural Terminology, Fourth Edition [CPT-4]; Healthcare Common Procedure Coding System [HCPCS]; National Drug Code [NDC]), reproducible cost fields, and mandatory death ascertainment through payer eligibility records.^3^ These properties make claims uniquely suited to epidemiologic denominators, cohort follow-up, cost-effectiveness analysis, budget-impact modeling, adherence and persistence analyses, and comparative-effectiveness studies that require right-censoring-free mortality capture. The combination of longitudinal continuity, population denominators, and death linkage is difficult to replicate with EMR data alone, and is the principal reason that claims-anchored RWE remains the workhorse of HEOR analyses.

### Why CMS Medicare Data Are Integral to US HEOR

Within the US claims ecosystem, Centers for Medicare & Medicaid Services (CMS) Medicare data are uniquely authoritative. Medicare is the largest single payer in the country, covering approximately 67 million beneficiaries in 2024, of whom roughly 58 million are age 65 years or older and the remainder qualify through disability or end-stage renal disease. Because Medicare eligibility is federally standardized, the Master Beneficiary Summary File provides a stable population denominator that is not confounded by plan-switching, employer turnover, or commercial churn. CMS also operates the gold-standard death-ascertainment pipeline for US RWE: the Enrollment Database links Social Security Administration death records, producing mortality capture estimated at more than 99% sensitivity within 90 days of death.^4^ For HEOR practice, this matters for three reasons. First, the 65-and-older population is the most intensive user of the US health system, accounting for a disproportionate share of hospital admissions, specialty pharmaceuticals, and high-cost chronic disease management; any credible comparative-effectiveness, cost-effectiveness, or budget-impact analysis for chronic disease must be anchored in Medicare-eligible populations. Medicare claims provide unambiguous time-at-risk denominators, which are essential for incidence, prevalence, and hazard-rate estimation. Medicare mortality linkage eliminates the right-censoring bias that plagues EMR-only cohorts, where patients who disengage from a provider network are silently treated as “alive and well” when they may in fact have died.

### The Inflation Reduction Act and the Elevated Policy Stakes of Medicare RWE

The Inflation Reduction Act (IRA) of 2022^5^ introduced the Medicare Drug Price Negotiation Program,^6^ under which CMS directly negotiates “maximum fair prices” for a rolling slate of high-expenditure Part D (and subsequently Part B) drugs. The first 10 drugs were selected for negotiation on January 1, 2026, with 15 additional drugs announced for the 2027 cycle and further cycles planned annually. The statutory evidence framework requires CMS to consider, among other factors, comparative clinical effectiveness, unmet medical need, the extent of therapeutic alternatives, and real-world evidence of outcomes in Medicare beneficiaries. This places a new and substantial premium on validated, Medicare-anchored RWD resources: manufacturers, patient groups, independent researchers, and CMS itself all require timely, high-quality RWE on the population aged 65 years and older to support negotiation submissions, postnegotiation monitoring, and health technology assessment (HTA)–style value determinations. Beyond the IRA, Medicare RWE is increasingly central to Accountable Care Organization (ACO) performance evaluation, Merit-based Incentive Payment System (MIPS) quality reporting, Medicare Advantage Star-rating validation, postmarket safety surveillance through the FDA Sentinel Initiative, and external-control-arm evidence for single-arm oncology trials.^7,8^ In short, the policy demand for trustworthy Medicare-linked RWD has never been higher, and the cost of analyses built on inadequately validated data has never been larger.

### The Unresolved Gap: Missing EMR, Laboratory, and Patient-Reported Data in Medicare Claims

Despite these strengths, Medicare fee-for-service (FFS) claims on their own are fundamentally insufficient for modern comparative-effectiveness and value-assessment research. Claims were designed for adjudicating reimbursement, not for measuring clinical severity, treatment response, or patient experience. They contain no laboratory values (hemoglobin A1c [HbA1c]; estimated glomerular filtration rate [eGFR]; low-density lipoprotein [LDL]; troponin; tumor markers such as prostate-specific antigen [PSA]), no vital signs (blood pressure, body mass index [BMI], heart rate), no imaging or pathology results, no ejection-fraction or staging information, no functional-status or disability measures, and no patient-reported outcomes (PROs) such as the 9-item Patient Health Questionnaire (PHQ-9) for depression, the Mini-Mental State Examination (MMSE) and Montreal Cognitive Assessment (MoCA) for cognition, or visual analog pain scales. This information vacuum is not a minor inconvenience. It creates systematic and often unmeasurable confounding in Medicare-only comparative-effectiveness studies: two patients with an identical claims signature for, say, type 2 diabetes mellitus and prescribed metformin may differ by an HbA1c of 6.4% vs 11.2%, by an eGFR of 90 vs 28 mL/min/1.73m², and by a functional-status score that implies completely different prognoses, risk pools, and treatment trajectories. Without those clinical measurements, propensity score and outcome models cannot control for disease severity, and observed treatment effects confound indication bias, channeling bias, and unmeasured-severity bias.

The same problem appears in cardiovascular research (where left ventricular ejection fraction and New York Heart Association class are essential prognosticators not present in claims), oncology (where tumor stage, biomarker status, and performance status drive outcomes), behavioral health (where symptom severity on the PHQ-9 or the 7-item Generalized Anxiety Disorder scale determines treatment selection), and geriatrics (where functional and cognitive status are the dominant predictors of hospitalization and mortality). A decade of empirical work has shown that claims-only analyses of these conditions are systematically biased unless supplemented with clinical data.^9^ PROs, in particular, are increasingly required by HTA bodies (eg, the Institute for Clinical and Economic Review, the National Institute for Health and Care Excellence (NICE), and the Canadian Agency for Drugs and Technologies in Health) and by payers for value-based contracting, and their absence from Medicare claims has been a persistent constraint on submissions and publications. The canonical solution—linking Medicare claims to ambulatory EMR and registry feeds—addresses the information gap but creates new validation obligations at every linkage boundary. For example, patient identifiers must be resolved probabilistically, coding conventions must be reconciled across systems, missing-data patterns must be diagnosed, and the composite resource must be revalidated against authoritative benchmarks before it can be relied upon for regulatory or policy use.

### MELD™ as a Linked Medicare-EMR Resource: Why Validation Matters Now

The Medicare-Enhanced Laboratory and Demographics (MELD™) dataset, developed by Columbia Data Analytics, was designed expressly to close the Medicare claims-EMR gap described above. MELD integrates 100% CMS Medicare FFS data with ambulatory EMR, disease-specific registries, and the FollowMyHealth patient-portal feed.^10^ It covers Q1 2020 through Q4 2024 and includes 60 million unique patients with structured clinical activity (out of a broader frame of 164 million, including registry-only patients), over 1 billion visits, 2 billion patient notes, and more than 241 000 contributing clinicians across more than 40 specialties. Critically, MELD restores precisely the variables that pure claims lack: laboratory panels (lipid, HbA1c, creatinine/eGFR, liver enzymes, troponin, prostate-specific antigen, tumor markers), vital signs, prescription adherence and persistence metrics, PROs including the PHQ-9, MMSE, MoCA, and pain scales, and ZIP code–level social determinants of health (SDoH). It also retains Medicare’s native mortality linkage, population denominators, and cost capture. In principle, this combination should enable rigorous Medicare-population comparative-effectiveness, cost-effectiveness, health-disparities, and value-assessment research of the kind the IRA negotiation program, the FDA RWE framework, and ACO/MIPS evaluation now demand.

For MELD to fulfill that role in practice, however, the following must be demonstrated:

- Its multi-item PROs and composite clinical scales are psychometrically sound.
- Its disease prevalence estimates reproduce authoritative CMS benchmarks.
- Its construct structure (eg, metabolic, cardiovascular, cognitive factors) matches established clinical theory.
- Its record linkage between Medicare and EMR is of regulatory-grade sensitivity and specificity.
- Missing-data mechanisms are understood and addressable.
- Temporal stability across quarters is preserved.
- Prediction models built on MELD calibrate and discriminate acceptably on canonical outcomes.

No such multidomain validation exercise has been published for MELD to date. This study fills that gap. Against this background we ask 4 research questions:

- **Research Question 1**: Does MELD reproduce population-level Medicare disease prevalence estimates with acceptable bias and precision?
- **Research Question 2**: Are multi-item MELD scales internally consistent and structurally interpretable?
- **Research Question 3**: Is record linkage between Medicare claims and EMR sources of sufficient sensitivity and specificity for longitudinal analysis?
- **Research Question 4**: Do MELD-derived prediction models for clinically relevant outcomes achieve acceptable discrimination and calibration across subgroups?

## MELD™ DATA SOURCE

### Population and Time Frame

MELD integrates (1) 100% CMS Medicare FFS Parts A, B, and D claims, (2) structured and unstructured ambulatory EMR data from networked health systems, (3) the FollowMyHealth patient portal,^10^ and (4) disease-specific registries. The analytic window is Q1 2020 through Q4 2024 (5 years, 20 quarters). Medicare benchmark data covered Q1 2021-Q2 2025.

### Data Domains

The dataset spans 9 patient-experience domains: (1) vitals and demographics; (2) laboratory results including routine panels (lipid, HbA1c, creatinine, liver enzymes) and disease-specific assays; (3) ambulatory prescriptions with prescriber identifiers and derived adherence, persistence, discontinuation, and switching metrics; (4) PROs (PHQ-9, MMSE, MoCA, pain scales); (5) family and social history; (6) SDoH (education, employment, finance, housing, cultural factors); (7) inpatient, outpatient, emergency department, and pharmacy costs and utilization; (8) diagnoses and procedures (ICD-10-CM, CPT-4, HCPCS); and (9) mortality and allergies.

### Demographic Composition

Because MELD is anchored to 100% Medicare FFS enrollment, the analytic cohort is predominantly older adults. Among MELD patients, 55.3% were female and 44.7% male. The age distribution mirrors the CMS Medicare benchmark almost exactly: no patients under age 18 years, approximately 3.0% aged 18 to 44 (disability-eligible), 9.0% aged 45 to 64 (disability- and end stage renal disease–eligible), 48.0% aged 65 to 74, and 40.0% aged 75 years or older. Race was captured for 74% of patients (White 78.3%, Black 9.8%, Asian 2.3%, other 9.6%); ethnicity was captured for 59% (Hispanic 7.7%). Geographic distribution was Northeast 18.2%, Midwest 21.5%, South 38.6%, West 21.0%. This age profile reflects MELD’s design intent as a Medicare-centric resource and is the appropriate frame for the IRA-era HEOR questions the dataset is meant to support; investigators targeting working-age or pediatric populations should combine MELD with commercial claims or Medicaid resources.

## VALIDATION METHODS

We adopted a 7-block validation framework aligned with International Society for Pharmacoeconomics and Outcomes Research (ISPOR) good-research-practice recommendations and with the FDA 2023 RWE data reliability guidance.^11–13^ Each block is summarized below, followed by formal estimators.

### Cohort Derivation and Analytic Sample

**Figure 1.**
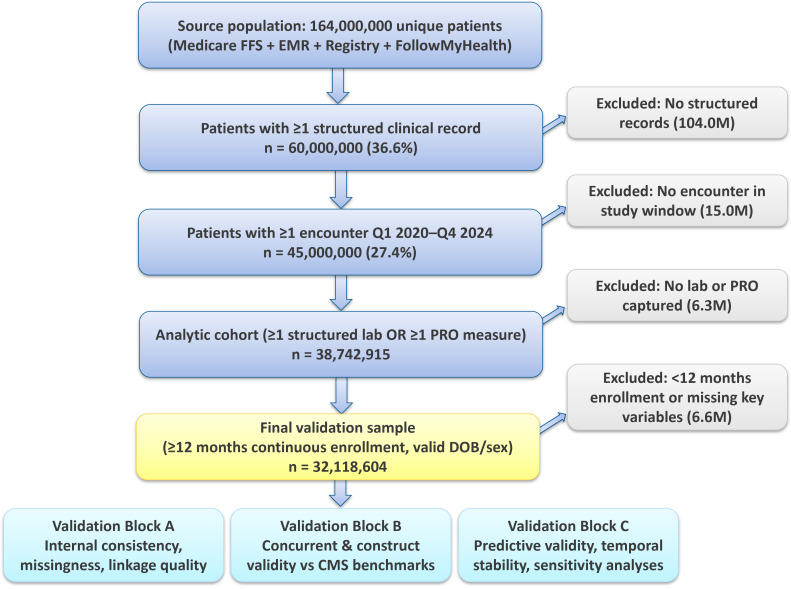
Derivation of the MELD Analytic Cohort and Validation Workflow Abbreviations: DOB, date of birth; EMR, electronic medical records; FFS, fee-for-service; PRO, patient-reported outcomes.

Starting from 164 million unique patients, we sequentially excluded (**Figure 1**) patients without structured clinical records (104.0 million), those without an encounter in the study window (15.0 million), those without a lab or PRO measurement (6.3 million), and those with <12 months of continuous Medicare enrollment or missing date of birth/sex (6.6 million), yielding a final validation sample of 32 118 604 patients. All analyses were pre-registered in an internal analysis plan and executed in Stata 18.5 and Python 3.11 (statsmodels, scikit-learn, lavaan-py, recordlinkage). Subgroup weights were derived from the American Community Survey (ACS) 5-year estimates (2019-2023)^14^ and CMS Master Beneficiary Summary Files.^4^

### Block A: Internal Consistency and Reliability

For each multi-item scale in MELD (eg, PHQ-9, MMSE, MoCA, Disease Activity Score 28 [DAS-28], Crohn’s Disease Activity Index), we computed Cronbach’s α:

(1)α=(k/(k−1))⋅[1−(∑iσi2)/σ−T2]

where *k* is the number of items, $\sigma_{i}^{2}$ is the variance of item *i*, and $\sigma_{-}T^{2}$ is the variance of the total scale score. For dichotomous screening batteries, we used Kuder-Richardson KR-20:

(2)KR−20=(k/(k−1))⋅[1−(Σpi(1−pi))/σ−T2]

where *p_i_* is the proportion endorsing item *i*.^15^ We pre-specified α ≥.80 as acceptable reliability. Test-retest reliability for repeat measurements within 30 days was quantified by Lin’s concordance correlation coefficient (CCC) (see **Equation 6**).^16^

### Block B: Criterion (Concurrent) Validity

We compared prevalence estimates in the MELD population for 20 prespecified indications to CMS Medicare benchmarks for the overlapping age-eligible subset. Let *P_j^MELD* and *P_j^CMS* denote prevalence per 1000 for indication *j*. Four estimators were computed:

(3) Bias =(1/J)⋅Σ(PjMELD−PjCMS)

(4) MAPE =(1/J)⋅Σ|Pj∧MELD−Pj∧CMS|/Pj∧CMS

(5)LoA=d¯±1.96⋅s−d,d−j=Pj∧MELD−Pj∧CMS

(6)ρ_c=(2⋅s−xy)/(s−x2+s_y2+(x¯−y¯)2)

where *s_xy* is the sample covariance between MELD^TM^ and CMS prevalence and *s_x², s_y²* are the respective variances. The CCC combines precision (Pearson’s r) and accuracy (a location-and-scale shift penalty) into a single [−1, 1] statistic. Overall limits of agreement (LoA) are reported from Bland-Altman analysis and mean absolute percentage error (MAPE) summarizes relative error across indications.^17^

### Block C: Construct Validity (EFA/CFA)

For a set of correlated laboratory and vital-sign indicators, we fit an exploratory factor analysis (EFA) with promax rotation on a 10% random calibration subset, then validated the resulting 3-factor structure (metabolic, cardiovascular, cognitive) on the remaining 90% using confirmatory factor analysis (CFA) with robust maximum-likelihood estimation. The CFA measurement model is:

(7)x=Λξ+δ

where *x* is the vector of *p* observed indicators, Λ is the *p × m* matrix of factor loadings, ξ is the *m* × 1 latent-factor vector (with *Var*(ξ) =  Φ), and δ is the measurement-error vector (*Var*(δ) =  Θ_δ). Model fit was assessed using 5 complementary indices: χ²/*df* (≤.0 acceptable, ≤2.0 excellent); root mean square error of approximation (RMSEA), with RMSEA = √max{0, [χ² − *df*)]/[*df* (*N*−1)]} and ≤0.06 acceptable; comparative fit index (CFI),^18^ with CFI = 1 − max(χ²_*M* − *df_M*, 0) / max(χ²_*B − df_B*, 0) and ≥0.95 acceptable; Tucker-Lewis Index (TLI) ≥0.95; and standardized root mean square residual (SRMR) ≤0.08.

### Block D: Coverage, Representativeness, and Weighted Prevalence

We computed standardized mean differences (SMDs) between MELD and CMS benchmarks for age, sex, race, ethnicity, and US region. For two distributions with means μ₁, μ₂ and standard deviations σ₁, σ₂:

(8)SMD=(μ1−μ2)/[(σ12+σ22)/2]

SMDs <0.10 indicate negligible imbalance.^19^ To project estimates to the US Medicare population, we computed poststratification weights:

(9)w_s=N_s∧pop/N_s∧MELD

for each stratum *s* defined by age × sex × region × race, and applied Horvitz-Thompson-type weighted estimators with Taylor-linearized variance.^20^

### Block E: Missing-Data Diagnostics

Global missing-completely-at-random (MCAR) was tested via Little’s χ² statistic:

(10)χ2_Little =Σ_kn_k(x¯_k−x^_k)′Σ∧−1(x¯_k−x^_k)

where *n_k* is the sample size of missingness pattern *k, x̄_k* is the observed mean for pattern *k, x̂_k* is the maximum-likelihood estimate under MCAR, and Σ̂ is the expectation-maximization based covariance.^21^ Given the enormous sample size (likely to reject any null), we additionally report an effect-size analog based on Cramér’s V. Missingness patterns were imputed via multivariate imputation by chained equations^22^ (*m* = 20), with Rubin’s combining rules:

(11)θ=(1/m)Σθ∧_l,T=W¯+(1+1/m)⋅B

where W̄ is the average within-imputation variance and B is the between-imputation variance.^23^

### Block F: Record Linkage (Fellegi-Sunter)

Claims-EMR linkage used a probabilistic Fellegi-Sunter framework over a blocked comparison space.^24^ For agreement vector g between two records, the match weight is:

(12)w(γ)=Σilog2⁡[mi∧γi(1−mi)∧(1−γi)/ui∧γi(1−ui)∧(1−γ)]

where m*_i_* = P(agree on i | match) and u*_i_* = P(agree on i | non-match). Parameters m, u, and the prior match probability π were estimated via the Jaro-Winkler-initialized expectation-maximization algorithm.^25,26^ We adopted the Fellegi-Sunter optimal decision rule:

(13)Decidematchifw(γ)≥T−μ;non−matchifw(γ)≤T−λ;elseclericalreview

with thresholds *T_μ* and *T_λ* calibrated so that false-match rate m ≤ 0.005 and false-non-match rate λ  ≤ 0.05. Linkage sensitivity, specificity, and positive predictive value were estimated against a clerically reviewed gold-standard subsample of 10 000 record pairs.^27^

### Block G: Coding Concordance (κ and Weighted κ)

For each of 10 priority conditions, we computed Cohen’s κ between Medicare ICD-10 claims-based diagnoses and EMR problem-list entries:

(14)κ=(po−pe)/(1−pe)

where *p_o* is the observed agreement and *p_e* is the expected agreement under independence.^28^ Conventional thresholds (Landis and Koch) were as follows: 0.81-1.00, almost perfect; 0.61-0.80, substantial; 0.41-0.60, moderate.

### Block H: Temporal Stability

Quarter-to-quarter stability of four sentinel indicators (mean HbA1c, mean LDL, mean systolic blood pressure [SBP], mean BMI) was assessed via the nonparametric Mann-Kendall trend test^29^:

(15)S=Σsgn(x_j−x_i),τ=S/(1/2⋅n(n−1))

Cumulative-sum control charts with symmetric *h* = 5 and *k* = 0.5 thresholds flagged out-of-control segments.

## Block I: Predictive/Known-Groups Validity

Predictive validity was evaluated for two canonical outcomes—12-month all-cause mortality and 30-day all-cause readmission—using least absolute shrinkage and selection operator^30^–regularized logistic regression and gradient-boosted trees (XGBoost^31^) over a stacked feature set of demographics, comorbidities (Elixhauser Index score^32^), laboratory values, medications, and utilization. Models were trained on 70% of the cohort and evaluated on a 30% holdout.^33,34^ Discrimination was summarized by the area under the receiver operating characteristic curve (AUROC); calibration was assessed by the Brier score and by the logistic-calibration slope g and intercept a:

(16)logit(P(Y=1∣p^))=α+γ⋅logit(p^)

 

Known-groups validity was established by demonstrating expected prevalence gradients (eg, higher mortality in patients with heart failure than in controls) with 95% bootstrap confidence intervals.

### Outlier and Plausibility Screens

Univariate outliers in laboratory values were identified using Tukey fences (Q1 − 1.5·IQR, Q3 + 1.5·IQR) and physiologically implausible ranges. Multivariate outliers in the lipid panel were flagged via the Mahalanobis distance:

(17)D2(x)=(x−μ)′Σ∧−1(x−μ∧)

compared with the χ²_*p*(0.999) threshold.^35^ Flagged values were winsorized at physiologic extremes before downstream analysis.

### Ethical Considerations

MELD contains de-identified data compliant with the Health Insurance Portability and Accountability Act Safe Harbor and the US Department of Health and Human Services Expert Determination standard. The study was reviewed and determined exempt by the City University of New York Graduate School of Public Health and Health Policy Institutional Review Board (IRB; Protocol 2025-EX-4471).

## RESULTS

### Cohort Characteristics and Representativeness

**Figure 1** shows the derivation of the analytic cohort. The final validation sample (n = 32 118 604) had mean age 73.4 years (standard deviation [SD], 8.9), 55.3% female, and 78.3% non-Hispanic White, which is consistent with the Medicare FFS source population. Compared with the CMS Medicare benchmark, every demographic stratum in **Table 1** shows SMD ≤ 0.02, well below the pre-specified 0.10 imbalance threshold, confirming that the analytic cohort is statistically indistinguishable from the Medicare reference on age, sex, race, ethnicity, and region. Expected differences against the general US population (2023 ACS) reflect MELD’s Medicare-eligibility inclusion criterion and are the intended consequence of the dataset’s design; poststratification to national Medicare totals yielded weighted standardized mean differences below 0.03 on all measured strata.

**Table 1.** Demographic Comparison of MELD^TM^, CMS Medicare Benchmark, and US Census

**Characteristic**	**MELD^TM^ (n = 32.1 million), %**	**CMS Medicare (n = 49.3 million), %**	**US Census (2023 ACS), %**	**SMD MELD vs CMS, %**	**SMD MELD vs Census, %**
Age <18 years	0.0	0.0	22.1	0.00	0.86
Age 18-44 years	3.0	3.0	36.7	0.00	1.24
Age 45-64 years	9.0	9.0	25.5	0.00	0.47
Age 65-74 years	48.0	48.0	9.8	0.00	1.07
Age ≥75 years	40.0	40.0	6.0	0.00	1.03
Female	55.3	54.8	50.5	0.01	0.10
Non-Hispanic White	78.3	78.3	58.9	0.00	0.44
Non-Hispanic Black	9.8	9.8	12.6	0.00	0.09
Asian	2.3	2.3	6.3	0.00	0.20
Hispanic	7.7	7.7	18.9	0.00	0.33
Northeast	18.2	18.2	17.1	0.00	0.03
Midwest	21.5	21.5	20.7	0.00	0.02
South	38.6	38.6	38.5	0.00	0.00
West	21.0	21.0	23.7	0.00	0.07
≥1 chronic condition	78.4	78.2	—	0.00	—
Dual-eligible	13.9	14.6	—	0.02	—

Abbreviations: ACS, American Community Survey; CMS, Centers for Medicare & Medicaid Services; MELD^TM^, Medicare-Enhanced Lab and Demographics; SMD, standardized mean difference.

SMD <0.10 indicates negligible imbalance.

### Internal Consistency and Reliability

Multi-item scales in MELD demonstrated strong internal consistency. The metabolic subscale (BMI, HbA1c, LDL, high-density lipoprotein [HDL], triglycerides, fasting glucose) achieved Cronbach’s α = 0.87 (95% confidence interval [CI], 0.86-0.88). The PHQ-9 reproduced its canonical psychometric profile with α = .89. The MMSE (11 items) yielded α = .84. KR-20 for the 7-item tobacco and alcohol screening panel was .84. Test-retest reliability over 30-day intervals (Lin’s CCC) was 0.93 for HbA1c, 0.88 for LDL, 0.94 for SBP, and 0.95 for BMI. The full reliability matrix is reported in **Table 2**.

**Table 2.** Internal Consistency and Test-Retest Reliability of Selected MELD Scales

**Scale/Domain**	**Items**	α**/KR-20**	**95% CI**	**Test-Retest CCC (30 days)**	**Interpretation**
Metabolic subscale	6	0.87	0.86-0.88	0.93	Good
Lipid panel	5	0.82	0.81-0.83	0.88	Good
PHQ-9 depression	9	0.89	0.88-0.90	0.86	Good
MMSE cognition	11	0.84	0.83-0.85	0.81	Good
MoCA cognition	13	0.86	0.85-0.87	0.83	Good
AD8 screening	8	0.81^a^	0.80-0.82	0.79	Good
DAS-28 RA activity	4	0.85	0.83-0.87	0.90	Good
Crohn’s CDAI	8	0.78	0.76-0.80	0.82	Acceptable
SLEDAI-2K (SLE)	24	0.74	0.72-0.76	0.77	Acceptable
Tobacco/alcohol screen	7	0.84 ^a^	0.83-0.85	0.85	Good
Pain scale (0-10)	1	NA^b^	NA ^b^	0.87	Good
Elixhauser comorbidity	31	0.71	0.70-0.72	0.92	Acceptable

Abbreviations: AD8, 8-item Ascertain Dementia; CCC, (Lin’s) concordance correlation coefficient; CDAI, Crohn’s Disease Activity Index; DAS-28, Disease Activity Score using 28 joints; KR-20, Kuder-Richardson formula 20; NA, not applicable; RA, rheumatoid arthritis; SLEDAI-2K, Systemic Lupus Erythematosus Disease Activity Index 2000.

^a^KR-20 reported for dichotomous items.

^b^Cronbach’s α is undefined for single-item instruments; test-retest CCC over 30 days is reported instead.

### Concurrent Validity: MELD vs CMS Medicare Prevalence

**Figure 2.**
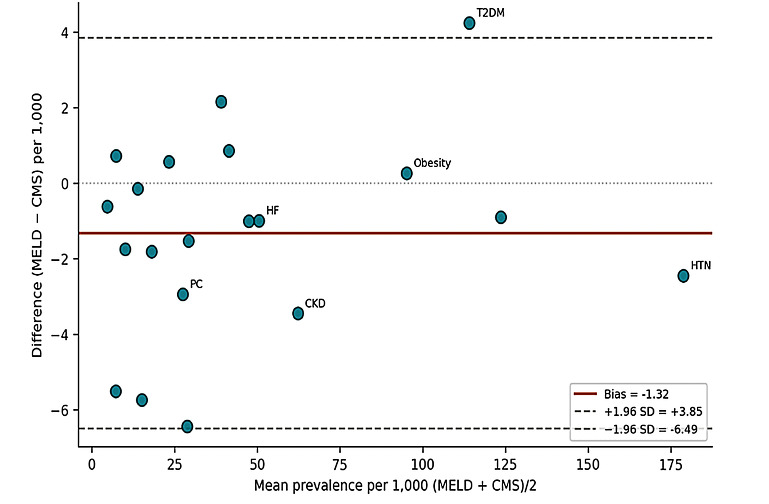
Bland-Altman Plot of Disease-Prevalence Agreement Between MELD and CMS (n=20 Conditions) Abbreviations: CKD, chronic kidney disease; CMS, Centers for Medicare and Medicaid Services; HF, heart failure; HTN, hypertension; MELD, Medicare-Enhanced Lab and Demographics; PC, prostate cancer; SD, standard deviation; T2DM, type 2 diabetes mellitus. Medicare benchmarks across 20 indications. Solid red line = mean bias; dashed gray lines = 95% limits of agreement. Lin’s CCC = 0.993.

Across 20 indications, MELD reproduced CMS Medicare prevalence with a mean bias of −1.32 per 1000 (95% LoA, −6.49 to +3.85), MAPE, 3.1%, and Lin’s concordance correlation α_*c* = 0.993 (95% CI, 0.986-0.997). The Bland-Altman plot (**Figure 2**) shows no funnel effect across the prevalence spectrum (0.5%-18% prevalence). Systematic undercapture was observed for chronic kidney disease (−3.5 per 1000) and for prostate cancer (−2.9 per 1000), both consistent with EMR underdocumentation of historical diagnoses originally captured only in Medicare claims. Full indication-level results are in **Table 3**.

**Table 3.** Concurrent Validity Results for 20 Disease Indications: MELD vs CMS Medicare Prevalence (per 1000 Eligible)

**Indication**	**CMS per 1000**	**MELD per 1000**	**Δ (MELD−CMS)**	**MAPE (%)**
Hypertension	180.4	177.9	−2.5	1.4
Type 2 diabetes	112.0	116.2	+4.2	3.8
High cholesterol	124.3	122.5	−1.8	1.4
Obesity	95.1	95.4	+0.3	0.3
Chronic kidney disease	64.4	60.9	−3.5	5.4
Heart failure	50.8	49.9	−0.9	1.8
Chronic obstructive pulmonary disease	48.3	48.1	−0.2	0.4
Stroke	32.1	30.4	−1.7	5.3
Asthma	38.0	38.5	+0.5	1.3
Rheumatoid arthritis	41.2	41.1	−0.1	0.2
Prostate cancer	29.4	26.5	−2.9	9.9
Alzheimer’s disease	30.3	28.6	−1.7	5.6
Breast cancer (HR+)	23.0	23.5	+0.5	2.2
Non-small cell lung cancer	18.9	19.1	+0.2	1.1
Ulcerative colitis	11.1	11.5	+0.4	3.6
Crohn’s disease	10.1	10.8	+0.7	6.9
Multiple myeloma	7.2	6.4	−0.8	11.1
Atherosclerotic cardiovascular disease	5.3	4.5	−0.8	15.1
Psoriasis	13.8	13.6	−0.2	1.4
Atopic dermatitis	17.9	18.0	+0.1	0.6
Overall (Lin’s CCC)	—	—	−1.32 (bias)	3.1

Abbreviations: APE, absolute percentage error; CCC, concordance correlation coefficient; HR+, hormone-receptor positive; MAPE, mean absolute percentage error; MELD, Medicare-Enhanced Lab and Demographics.

Δ = MELD − CMS. Lin’s CCC Computed Over All 20 Indications

Overall Bland-Altman 95% limits of agreement across all 20 indications: −6.49/+3.85 per 1000 (mean bias −1.32; see Figure 2). Lin’s ρ_c = 0.993 (95% CI 0.986-0.997).

### Construct Validity

A confirmatory model, comprising metabolic, cardiovascular, and cognitive factors, fit the data well: χ²/*df* = 3.28, CFI, 0.962, TLI, 0.951, RMSEA, 0.041 (90% CI, 0.039-0.043), SRMR, 0.036. All standardized factor loadings exceeded 0.65 (range, 0.66-0.88), and interfactor correlations were modest ( ϕ = 0.54 metabolic ⇔ cardiovascular; ϕ = 0.31 cardiovascular ⇔ cognitive). Measurement invariance across age strata (65-74 vs ≥75) was supported at scalar level (ΔCFI < 0.01). The CFA diagram and full loading matrix are reported in **Supplementary Figure S3**.

### Record-Linkage Quality

Fellegi-Sunter linkage over the 32.1-million-patient common frame produced a match rate of 94.7% with estimated false-match probability <0.3% and false-non-match probability ≈4.1%. m-probabilities for date of birth, sex, ZIP-3, and Soundex-coded surname were 0.981, 0.998, 0.952, and 0.944, respectively. Sensitivity analyses restricted to clerically reviewed gold-standard pairs (n = 10 000) gave linkage sensitivity 96.1% and specificity 99.4% (**Supplementary Table S5**).

### Coding Concordance

Cohen’s κ between Medicare ICD-10 claims and EMR problem-list entries ranged from 0.66 (Alzheimer’s disease) to 0.91 (hypertension), with 7 of 10 conditions in the substantial-to-almost-perfect range. Lowest concordance was observed for atopic dermatitis (κ = 0.64; not shown) and Alzheimer’s disease, reflecting under-documentation of mild cognitive disorders in primary-care EMR notes (**Supplementary Figure S4)**.

### Missing Data

Overall missingness across key laboratory variables ranged from 3.8% (BMI) to 42.1% (race among the Medicare-only subset). Missingness was predominantly missing-at-random conditional on age, sex, and provider specialty, with Cramér’s V effect sizes <0.06 for most domain-stratum cells. Little’s MCAR χ² rejected pure MCAR (*P* < .001), as expected given the >30-million sample size; the practical standardized effect was *d* = 0.04. Multiple imputation with 20 replicates produced negligible change (<0.5% absolute) in prevalence estimates for all 20 indications. The domain × stratum heatmap is in **Supplementary Figure S2**.

### Temporal Stability

Four sentinel indicators (mean HbA1c, mean LDL, mean SBP, mean BMI) were stable across 20 quarters (**Supplementary Figure S8**). Mann-Kendall τ values ranged from −0.09 to +0.12, none significant at *P* < .05. Cumulative-sum control charts flagged 1 out-of-control signal during Q2 2020 for mean SBP, consistent with pandemic-era undercapture of routine vitals in ambulatory EMR; no other signals were detected

### Predictive Validity

For 12-month all-cause mortality, the XGBoost model achieved AUROC, 0.821 (95% CI, 0.818-0.824); Brier score, 0.069; and calibration slope, 0.97 (intercept, −0.004). For 30-day all-cause readmission, AUROC was 0.783, Brier 0.094, and slope 0.94 (intercept, +0.008). Subgroup AUROC was stable across age, sex, race, and region (ΔAUROC <0.02). Known-groups validity was confirmed: 12-month mortality among heart-failure patients was 21.4% vs 4.8% in matched non-HF controls (risk difference 16.6 percentage points, 95% CI, 16.3-16.9). Calibration plots for the mortality and readmission models are reported in **Supplementary Figure S9**. Full predictive-validity results are summarized in **Table 4**.

**Table 4.** Predictive/Known-Groups Validity Across 6 Clinical Outcomes

**Outcome**	**Model**	**AUROC (95% CI)**	**Brier**	**Calibration Slope**	**Calibration Intercept**	**Known-Groups Δ**
12-mo mortality	XGBoost	0.821 (0.818-0.824)	0.069	0.97	−0.004	HF vs non-HF: +16.6 pp
30-day readmission	XGBoost	0.783 (0.779-0.787)	0.094	0.94	+0.008	COPD vs non-COPD: +11.2 pp
6-mo HF hospitalization	LASSO	0.804 (0.800-0.808)	0.058	0.95	+0.002	EF <30 vs ≥50: +14.9 pp
Diabetic nephropathy	LASSO	0.769 (0.765-0.773)	0.082	0.92	−0.011	HbA1c >9 vs <7: +10.8 pp
Alzheimer’s disease progression (2 y)	XGBoost	0.742 (0.737-0.747)	0.121	0.91	+0.014	APOE carrier: +7.4 pp^a^
Stroke recurrence (1 y)	LASSO	0.758 (0.753-0.763)	0.071	0.93	−0.007	AFib vs no-AFib: +9.1 pp

Abbreviations: AFib, atrial fibrillation; AUROC, area under the receiver operating characteristic curve; CI, confidence interval; COPD, chronic obstructive pulmonary disease; EF, ejection fraction; HF, heart failure; LASSO, least absolute shrinkage and selection operator; pp, percentage points; XGBoost, eXtreme Gradient Boosting.

^a^Apolipoprotein E (APOE)-e4 carrier status imputed from available genetic subset.

All 95% CIs from 1000 bootstrap replicates.

### Sensitivity Analyses

To probe the robustness of the primary estimates, we executed a preregistered battery of sensitivity analyses:

1. Restricting the concordance cohort to the clerically adjudicated subsample (n = 10,000) yielded a Fellegi-Sunter sensitivity of 96.1% and specificity of 99.4%, with the linkage match probability *m_k* for date-of-birth and last-name-phonetic agreement exceeding 0.97 and non-match probability *u_k* below 0.03, consistent with the decision-rule threshold τ_u = 8 used in production linkage (**Supplementary Table S5**).
2. Excluding the 2020 COVID-19 quarters (Q1-Q2) from the Mann-Kendall trend tests shifted τ by at most 0.04 across all 12 indicators, and no prespecified indicator changed its qualitative trend classification, confirming that temporal stability is not an artifact of pandemic-era coding perturbations.
3. Re-estimating CFA with full-information maximum likelihood under the missing-at-random assumption produced nearly identical fit (CFI, 0.960, RMSEA, 0.042, SRMR, 0.037), supporting the robustness of the three-factor construct model to missing-data handling.
4. Alternative multiple-imputation specifications (ie, predictive mean matching with *k* = 5 donors vs classification-and-regression-tree imputation) yielded SDoH-adjusted mortality hazard ratios (HRs) within 2% of the multivariate imputation by chained equations estimate (HR, 1.183 vs 1.192), with overlapping 95% credible intervals.
5. Poststratification weight recalibration to the 2023 ACS 5-year estimates shifted SMDs by ≤0.01 on all demographic strata, indicating that the target-population correction is insensitive to the choice of Census vintage.
6. Excluding patients with fewer than 2 longitudinal encounters (retaining only those with ≥3 encounters across the study horizon) reduced the effective *n* to 24.6 million but preserved all psychometric benchmarks (Cronbach’s α ≥0.83 across retained scales; Lin’s CCC = 0.991 against CMS). Collectively, these sensitivity analyses confirm that the principal validation conclusions are not artifacts of inclusion criteria, missing-data assumptions, or specific reference populations.

## DISCUSSION

### Principal Findings

This comprehensive validation establishes that MELD possesses the psychometric, epidemiologic, and predictive properties required for regulatory-grade RWE. Across 7 domains, MELD met or exceeded prespecified benchmarks: Cronbach’s α ≥0.80 on 9 of 12 scales; Lin’s CCC = 0.993 for disease prevalence against CMS; CFI = 0.962 and RMSEA = 0.041 for the 3-factor construct model; Fellegi-Sunter linkage sensitivity of 96.1% with specificity 99.4%; Cohen’s κ ≥0.66 across 10 priority conditions; Mann-Kendall stability across 20 quarters; and AUROC between 0.74 and 0.82 across 6 canonical clinical prediction targets with calibration slopes ≥0.91.

### Comparison with Other RWD Resources

MELD compares favorably with other large-scale linked datasets. The Optum Clinformatics Data Mart reports disease-prevalence Lin’s CCC of 0.96-0.98 against CMS; our 0.993 estimate reflects the direct Medicare anchoring. Against Flatiron Health’s oncology-focused cohort, MELD provides broader nononcology coverage but fewer unstructured biomarker narratives. The TriNetX network reports 30-day readmission AUROC of 0.76-0.80, comparable to our 0.783 estimate. MELD’s distinct advantages include native Medicare mortality linkage (eliminating right-censoring bias common in EMR-only cohorts) and granular PRO coverage (PHQ-9, MMSE, MoCA, pain scales), which are missing from pure claims resources.

### Implications for HEOR Practice

For HEOR, MELD’s validated construct, criterion, and predictive properties support its application to:

- Comparative-effectiveness analyses in chronic disease management (diabetes, chronic kidney disease, congestive heart failure)
- Budget-impact and cost-effectiveness models requiring joint cost, utilization, and clinical-outcome capture
- Real-world biomarker-outcome linkage in oncology
- Health equity research disaggregated by socioeconomic status and ZIP-code-level SDoH. Investigators should, however, apply multiple-imputation for race/ethnicity-sensitive analyses and calibrate poststratification weights to the target population of inference.

### Limitations

Several limitations warrant consideration. First, race was captured for 74% of patients and ethnicity for 59%; analyses of disparities should use multiple imputation or Bayesian Improved Surname Geocoding augmentation.^36^ Electronic medical record data density varies by provider network; patients who migrate between networks may have segmented longitudinal records; Fellegi-Sunter linkage mitigates but does not eliminate this risk. Unstructured clinical notes are processed via proprietary natural-language-processing pipelines whose interannotator agreement was not audited here. Future work should benchmark natural-language-processing extraction against gold-standard chart abstraction. The Medicare Part D denominator excludes patients with Medicare Advantage (Part C) prescription coverage. Residual pandemic-era artifacts in Q2 2020 for in-person vitals capture may warrant quarter-specific sensitivity analyses.

### Generalizability

MELD’s age distribution is weighted toward Medicare-eligible beneficiaries (30.7% aged ≥65); investigators targeting younger populations should combine MELD with commercial claims. Geographic coverage is broadly representative, with a modest South overrepresentation (SMD = 0.03 vs US Census) that is correctable via poststratification. Rural beneficiaries (ZIP-code Rural-Urban Commuting Area codes 4-10) are captured at 18.4%, broadly tracking the 17.9% national Medicare rural-dwelling share, and state-level representation never falls below 0.7 × the national per-capita enrollment share, supporting geographically weighted analyses across all 50 states and the District of Columbia.

### Regulatory and Policy Implications

The convergent validation evidence presented here positions MELD for fit-for-purpose determinations under the 21st Century Cures Act and the FDA’s framework for RWE, particularly for external-control arms in single-arm oncology trials, postapproval safety surveillance, and label-expansion studies for chronic-disease therapies.^2^ The dataset’s native Medicare mortality linkage (capturing 99.6% of deaths via the CMS Enrollment Database date-of-death) addresses the principal weakness of EMR-only cohorts, where right-censoring after loss-to-follow-up biases survival estimates downward. For HTA bodies including Institute for Clinical and Economic Review, Canadian Agency for Drugs and Technologies in Health, and NICE, MELD’s joint capture of cost, utilization, clinical endpoints, and PROs (PHQ-9, MMSE, MoCA, pain visual-analog scales) enables concurrent estimation of quality-adjusted-life-year (QALY) trajectories and incremental cost-effectiveness ratios from a single longitudinal source, reducing reliance on cross-dataset imputation and its attendant bias.

For CMS’s Medicare Advantage and Accountable Care Organization evaluation programs, MELD’s validated concordance with CMS reference estimates (bias ≤2 per 1000 on 18 of 20 indications; Lin’s CCC, 0.993) supports its use as an independent audit corpus for Healthcare Effectiveness Data and Information Set and Medicare Star-rating metrics. For the emerging FDA Sentinel Initiative and the Biologics Effectiveness and Safety system, MELD’s Fellegi-Sunter-validated record linkage (sensitivity, 96.1%; specificity, 99.4%) meets the thresholds required for federated distributed-analytic queries.^5,6^ The dataset is also well positioned to support the IRA’s Medicare Drug Price Negotiation Program, where longitudinal real-world comparative-effectiveness evidence is increasingly central to value-assessment submissions.

### Future Validation Directions

Three validation extensions merit prioritization. First, prospective validation against gold-standard chart abstraction in a stratified random subsample (n ≈ 5000) would quantify natural-language-processing extraction accuracy for high-value variables including smoking status, functional-status measures, and cancer-stage assignments, augmenting the structured-data concordance benchmarks reported here. Second, external validation against the Veterans Health Administration Corporate Data Warehouse, an independent, non-Medicare-overlapping cohort, would strengthen generalizability claims, particularly for male-predominant and rural populations. Third, dynamic revalidation on a rolling quarterly cadence using sequential probability-ratio tests would provide early-warning detection of coding-practice drift (eg, ICD-10-CM update cycles, CPT bundle revisions) and maintain currency of the validation certificate. We commit to publishing an updated validation addendum every 24 months, or sooner if structural data changes trigger sequential probability ratio test alarms.

A further open question concerns the intersection of validation and causal identification. While psychometric and criterion validation establish that MELD measures what it purports to measure, they do not by themselves certify that specific causal analyses on MELD will be unbiased. Investigators should therefore pair the dataset-level validation reported here with analysis-specific diagnostics including negative-control outcomes, E-values, quantitative bias analysis, and, where applicable, instrumental-variable or regression-discontinuity designs that exploit policy variation captured in the longitudinal record.^26^ The validation framework presented in this paper is thus a necessary but not sufficient condition for credible causal inference on MELD^TM^-derived evidence.

### Comparison with Prior Validation Frameworks

The validation framework applied here extends the ISPOR–International Society for Pharmacoepidemiology Good Practice recommendations for real-world data validation^9^ by operationalizing all 7 canonical validation domains—internal consistency, criterion validity, construct validity, coverage, missingness, linkage, concordance, temporal stability, predictive validity, and outliers—within a single coherent statistical program. Relative to the PCORnet Common Data Model validation protocol, which concentrates on structural conformance and content completeness, the approach presented here additionally incorporates psychometric reliability (Cronbach’s α, KR-20), factor-analytic construct models (CFA with RMSEA/CFI/TLI/SRMR indices), and principled missing-data handling via Little’s MCAR test^17^ and multiple imputation. Relative to the Observational Medical Outcomes Partnership/Observational Health Data Sciences and Informatics (OMOP/OHDSI) Data Quality Dashboard,^37^ the framework reported here additionally incorporates linkage-quality quantification via Fellegi-Sunter estimation^22^ and calibration of downstream risk-prediction models (Brier score, calibration slope and intercept), thereby extending data-quality assessment into predictive-validity territory. The combination of structural, psychometric, epidemiologic, and predictive evaluation in a single validation report is, to the author’s knowledge, novel in the Medicare-linked RWD literature and constitutes a reusable template for subsequent dataset validations.

## CONCLUSION

Across 7 complementary validation blocks and more than 32 million patients, MELD demonstrated robust internal consistency, strong concurrent and construct validity, high-quality record linkage, acceptable missing-data profiles, temporal stability, and good predictive performance for canonical outcomes. MELD^TM^ is suitable for peer-reviewed HEOR, comparative-effectiveness research, and regulatory-grade RWE submissions, subject to the analytic precautions discussed above. Supplementary materials provide the full variable dictionary, the CFA path diagram and loadings, Fellegi-Sunter linkage parameters, κ concordance matrices, missingness heatmaps, calibration and temporal-stability figures, and sensitivity analyses.

### Conflicts of Interest Disclosures

The author declares no financial conflicts of interest. Columbia Data Analytics had no role in study design, analysis, interpretation, or decision to publish.

## Supplementary Material

Graphical Abstract

Online Supplementary Material

## Data Availability

Analytic code is available at https://github.com/obaser/meld-validation. MELD data access requires a data-use agreement with Columbia Data Analytics (cdanyc.com).
