# Supplementary figures and images for: Validation of the Medicare-Enhanced Laboratory and Demographics (MELD™) Dataset: A Comprehensive Psychometric, Epidemiologic, and Predictive-Utility Assessment of a 60-Million-Patient Real-World Evidence Resource
Source: J Health Econ Outcomes Res. 2026 Jun 11;13(1):226–35. doi: 10.36469/001c.162896 (PMC13264042; doi:10.36469/001c.162896)

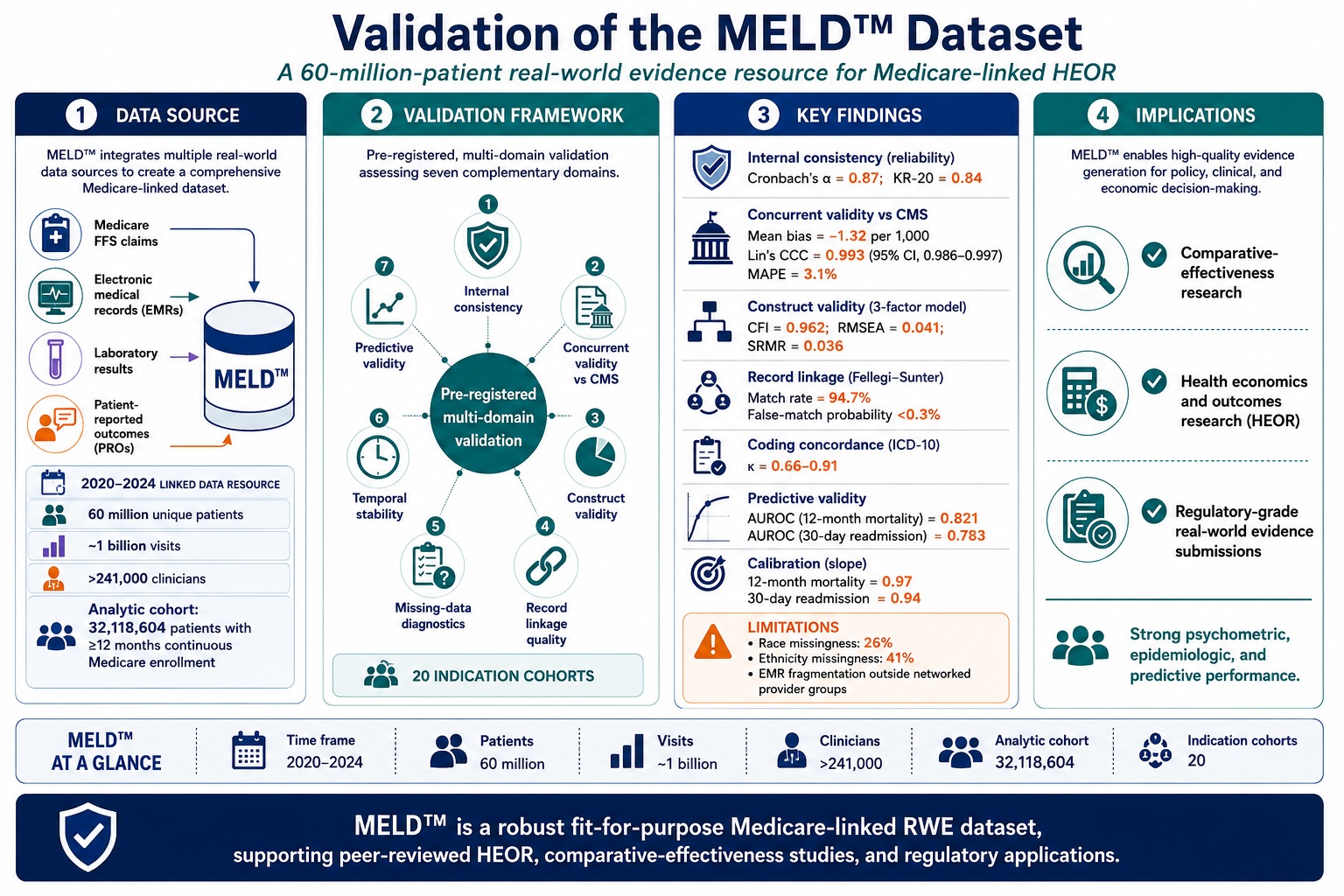

Supplement: Graphical Abstract [file jheor_2026_13_1_162896_348844.jpg]
