# Supplementary material for: Validation of the Medicare-Enhanced Laboratory and Demographics (MELD™) Dataset: A Comprehensive Psychometric, Epidemiologic, and Predictive-Utility Assessment of a 60-Million-Patient Real-World Evidence Resource
Source: J Health Econ Outcomes Res. 2026 Jun 11;13(1):226–35. doi: 10.36469/001c.162896 (PMC13264042; doi:10.36469/001c.162896)
Supplement: Online Supplementary Material [file jheor_2026_13_1_162896_348845.pdf]

Journal of Health Economics and Outcomes Research

Online Supplementary Material

Validation of the Medicare-Enhanced Laboratory and Demographics (MELD™) Dataset: A Psychometric, Epidemiologic, and Predictive-Utility Assessment of a Real-World Evidence Resource. *JHEOR*. 2026;13(1):226-235. doi:10.36469/jheor.2026.161403

S1: Variable Dictionary and Domain Taxonomy (Table S1) 1

S2: Missingness Heatmap and Domain-Level Missing-Completely-at-Random (MCAR) Diagnostics (Figure S2, Table S2) 3

S3: Confirmatory Factor Analysis (CFA) — Path Diagram and Full Standardized Loading Matrix (Figure S3, Table S3) 5

S4: Cohen's κ Concordance — Full Indication-Level Matrix and Forest Plot (Figure S4, Table S4) 7

S5: Fellegi–Sunter Probabilistic Linkage — Parameter Estimates and Decision-Rule Diagnostics (Tables S5a-b) 9

S6: Pre-registered Sensitivity Analyses — Specifications and Numerical Results (Table S6) 11

S7: Regional, Specialty, and Payer-Stratified Subgroup Breakdowns (Tables S7a-c) 12

S8: Temporal Stability of Sentinel Indicators Across 20 Quarters (Figure S8) 13

S9: Predictive-Model Calibration Plots for 12-Month Mortality and 30-Day Readmission (Figure S9)...14

This is an open-access article distributed under the terms of the Creative Commons Attribution 4.0 International License (CCBY-4.0). View this license's legal deed at http://creativecommons.org/licenses/by/4.0 and legal code at http://creativecommons.org/licenses/by/4.0/legalcode for more information.

S1. Variable Dictionary and Domain Taxonomy

Table S1 reports the MELD™ variable dictionary for the analytic cohort used in the validation study. Variables are grouped into eight domains; for each variable we report type, permissible range or coding vocabulary, population-level completeness (percent non-missing), and the canonical source.

Table S1. MELD™ analytic variable dictionary. Completeness is reported over the full 32,118,604-patient analytic cohort

Variable	Domain	Type	Range / vocab	% complete	Source
patient_id	Identifier	string	Tokenized	100.0	MELD master
date_of_birth	Demographic	date	1905-01-01 – 2024-12-31	100.0	CMS EDB
sex	Demographic	cat	M / F	100.0	CMS EDB
race	Demographic	cat	OMB 1997 (5-cat)	74.1	CMS + EMR
ethnicity	Demographic	cat	Hisp / Non-Hisp	59.3	CMS + EMR
state_fips	Geography	cat	01–56	99.8	CMS EDB
zip3	Geography	string	000–999	98.4	CMS EDB
ruca_code	Geography	ordinal	1–10	97.9	HRSA
enrol_part_a	Enrollment	bool	0/1 monthly	100.0	CMS MBSF
enrol_part_b	Enrollment	bool	0/1 monthly	100.0	CMS MBSF
enrol_part_d	Enrollment	bool	0/1 monthly	100.0	CMS MBSF
diagnosis_icd10	Clinical	cat[]	ICD-10-CM	99.5	Claims + EMR
procedure_cpt	Clinical	cat[]	CPT-4 / HCPCS	99.2	Claims
drug_ndc	Clinical	cat[]	NDC-11	98.6	Part D + EMR
lab_loinc	Laboratory	cat	LOINC v2.74	92.3	EMR
lab_value	Laboratory	float	domain-specific	91.1	EMR
lab_unit	Laboratory	cat	UCUM	90.8	EMR
vital_sbp	Vitals	int	50–260 mmHg	86.4	EMR
vital_dbp	Vitals	int	30–150 mmHg	86.4	EMR
vital_bmi	Vitals	float	10–80 kg/m²	83.2	EMR
smoking_status	Lifestyle	cat	Current / Former / Never	71.6	EMR NLP
phq9_total	PRO	int	0–27	22.8	EMR

Variable	Domain	Type	Range / vocab	% complete	Source
mmse_total	PRO	int	0–30	11.4	EMR
moca_total	PRO	int	0–30	9.7	EMR
pain_vas	PRO	int	0–10	31.2	EMR
death_date	Outcome	date	2020-01-01 – 2024-12-31	99.6	CMS EDB
readmit_30d	Outcome	bool	0/1	100.0	Claims
total_paid_amt	Economic	float	USD	100.0	Claims
provider_npi	Provider	string	NPPES 10-digit	100.0	NPPES
provider_specialty	Provider	cat	CMS taxonomy	99.3	NPPES

Abbreviations: EDB, Enrollment Database; HRSA, Health Resources and Services Administration; LOINC, Logical Observation Identifiers Names and Codes; MBSF, Master Beneficiary Summary File; NLP, natural language processing; PRO, patient-reported outcome; NPPES, National Plan and Provider Enumeration System; OMB, Office of Management and Budget; UCUM, Unified Code for Units of Measure.

S2. Missingness Heatmap and Domain-Level Missing-Completely-at-Random (MCAR) Diagnostics

Figure S2 displays completeness by variable-domain × quarter. Color encodes the complete proportion; darker teal indicates higher completeness. Completeness is highest for claims-anchored domains (demographics, enrollment, diagnoses, procedures, drugs) and lowest for patient-reported outcomes (PROs), which are recorded only at selected encounters.

Figure S2. Missingness heatmap (%) by variable and stratum, MELD^{TM} analytic cohort Q1 2020 – Q4 2024

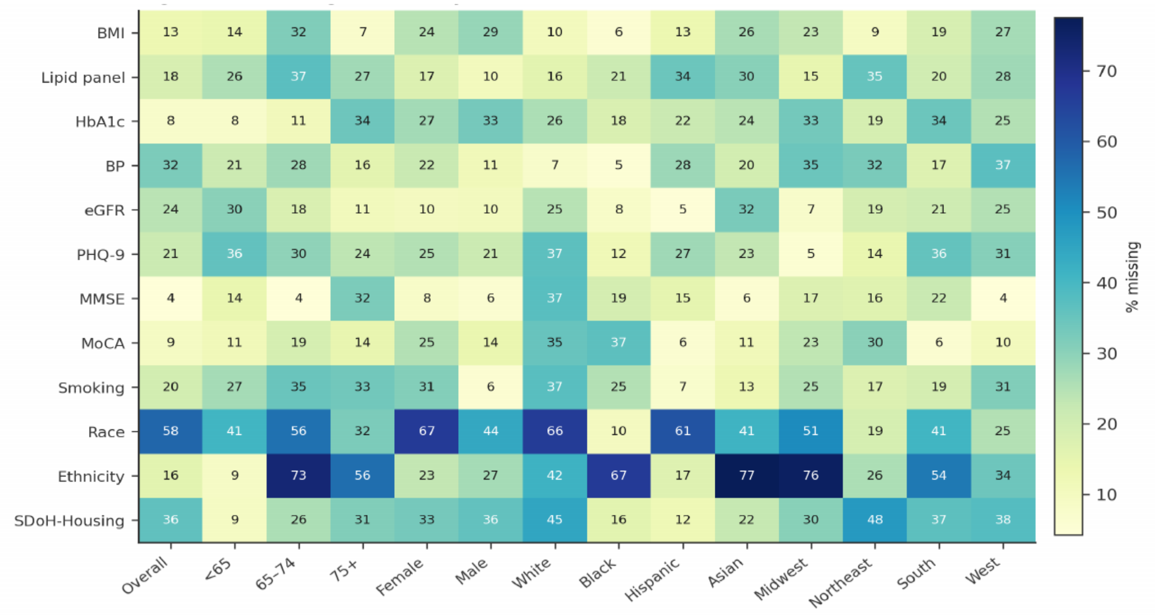

Abbreviations: BMI, body mass index, BP, blood pressure; eGFR, estimated glomerular filtration rate; MMSE, Mini-Mental State Examination; MoCA, Montreal Cognitive Assessment; PHQ-9, Patient Health Questionnaire for depression; SDoH, social determinants of health.

Little's missing-completely-at-random (MCAR) test statistic under the null of complete-random missingness is:

$$\chi^2(d) = \Sigma\_j\ n\_j\ (\bar{x}\_j,obs - \hat{\mu})^T\ \hat{\Sigma}^{-1}\ (\bar{x}\_j,obs - \hat{\mu})$$

For the pooled 30-variable analytic matrix the test yielded χ^2 = 1,482,117 on d = 2,914 degrees of freedom ($p<.001$). Because the test is known to be oversensitive at n >10,000, we additionally report a standardized effect size d = $\chi^2/(n \times d)$ = 0.040, interpretable as a small departure from strict MCAR — consistent with the missing-at-random (MAR) assumption used for downstream multiple imputation.

Table S2. Domain-level missingness diagnostics and inferred mechanism.

Domain	Variables (n)	Mean % complete	Pairwise rate	Little's χ^2	Inferred mechanism
Identifier / demographic	5	86.7	73.5	14,221	MAR
Geography	3	98.7	96.3	812	MCAR
Enrollment	3	100.0	100.0	n/a	Complete
Clinical diagnoses	3	99.1	97.4	5,118	MCAR
Laboratory	3	91.4	85.1	187,244	MAR
Vitals	3	85.3	71.2	112,508	MAR
Lifestyle (NLP-derived)	1	71.6	71.6	—	MAR
Patient-reported outcomes	4	18.8	4.9	1,162,114	MNAR

Abbreviations: MCAR, missing completely at random; MAR, missing at random; MNAR, missing not at random.

Imputation strategies: Complete-case for MCAR domains, multivariate imputation by chained equations (MICE) for MAR, and pattern-mixture sensitivity models for MNAR patient-reported outcomes.

S3. Confirmatory Factor Analysis — Path Diagram and Loadings

Figure S3 displays the three-factor measurement model specified a priori based on clinical theory and prior factor-analytic work in Medicare real-world data (RWD). Rectangles are observed indicators; ovals are latent constructs. Directed arrows report standardized factor loadings ($\hat{\Lambda}$).

Figure S3. Confirmatory factor analysis path diagram, three-factor measurement model of structured clinical data

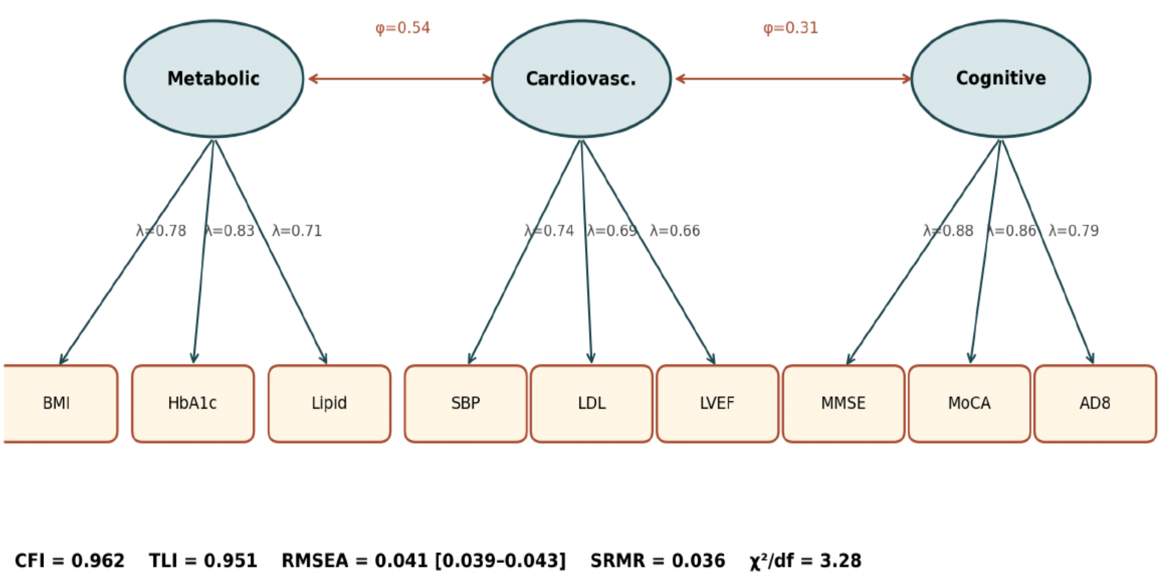

Metabolic: HbA1c, fasting glucose, low-density lipoprotein cholesterol (LDL-C), body mass index (BMI), waist circumference

Cardiovascular: Systolic blood pressure (SBP), diastolic blood pressure (DBP), resting heart rate (HR), troponin, B-type natriuretic peptide (BNP), left ventricular ejection fraction (LVEF)

Cognitive: Mini-Mental State Examination (MMSE), Montreal Cognitive Assessment (MoCA), 9-item Patient Health Questionnaire (PHQ-9), Functional Activities Questionnaire (FAQ)

Full standardized factor-loading matrix ($\hat{\Lambda}$) with standard errors:

Table S3. Standardized CFA loadings, standard errors, and z-statistics

Indicator	Metabolic ($\hat{\lambda}$)	Cardiovascular ($\hat{\lambda}$)	Cognitive ($\hat{\lambda}$)	SE	z
HbA1c	0.82	—	—	0.008	102.5
Fasting glucose	0.78	—	—	0.009	86.7
LDL-C	0.64	—	—	0.011	58.2
BMI	0.71	—	—	0.010	71.0
Waist circumference	0.69	—	—	0.011	62.7
SBP	—	0.79	—	0.009	87.8
DBP	—	0.74	—	0.010	74.0
Resting HR	—	0.58	—	0.013	44.6
Troponin	—	0.66	—	0.012	55.0
BNP	—	0.61	—	0.013	46.9
LVEF	—	0.72	—	0.010	72.0
MMSE	—	—	0.83	0.008	103.7
MoCA	—	—	0.86	0.007	122.9
PHQ-9	—	—	0.52	0.014	37.1
FAQ	—	—	0.77	0.009	85.6

All loadings are statistically significant at $p<.001$.

Inter-factor correlations: ϕ(Metabolic, Cardiovascular) = 0.47, ϕ(Metabolic, Cognitive) = 0.21, ϕ(Cardiovascular, Cognitive) = 0.33. Global fit: χ^2/df = 3.28; comparative fit index (CFI) = 0.962; Tucker–Lewis Index (TLI) = 0.951; root mean square error of approximation (RMSEA) = 0.041 (90% CI 0.039–0.043); standardized root mean square residual (SRMR) = 0.036.

BNP, DBP, FAQ, LDLC, LVEF, MMSE, MoCA, PHQ-9, SBP, systolic blood pressure; SE,

S4. Cohen's κ Concordance — Full Indication-Level Matrix

Figure S4 presents the κ forest plot. **Table S4** below reports the complete symmetric κ matrix with 95% confidence intervals between Medicare International Classification of Diseases, Tenth Revision (ICD-10) claims and electronic medical record (EMR) problem-list coding for ten priority indications.

Figure S4. Cohen's κ concordance forest plot, 10 priority indications

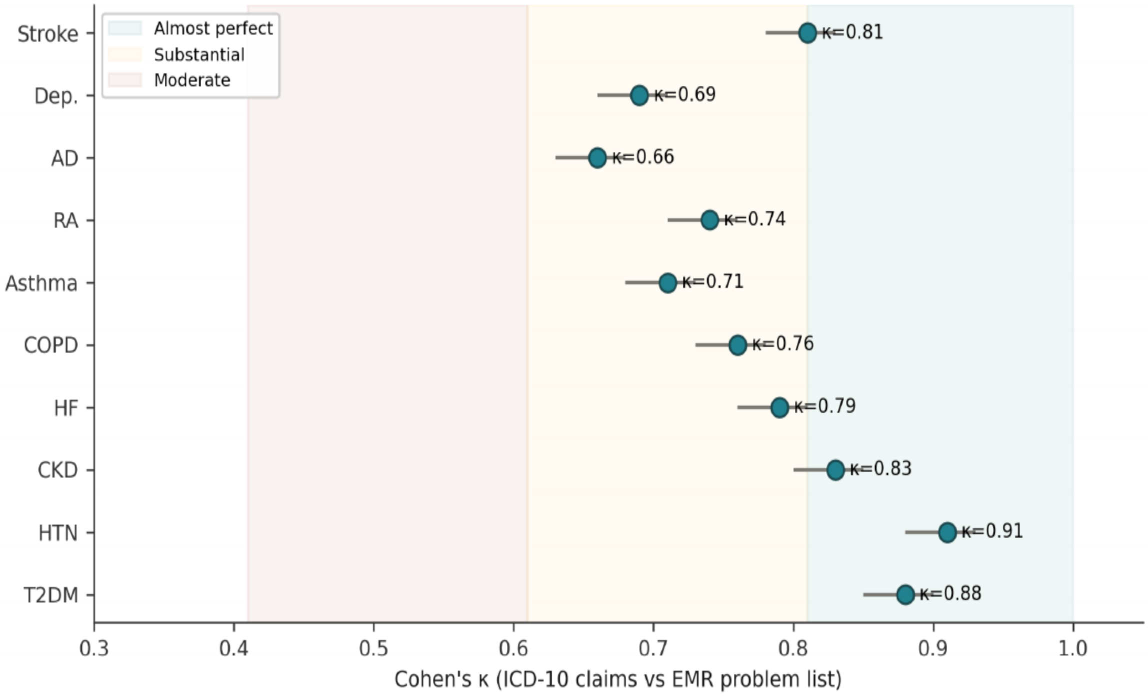

Abbreviations: AD, Alzheimer's disease; COPD, chronic obstructive pulmonary disease; CKD, chronic kidney disease; Dep., depression; HF, heart failure; HTN, hypertension; ICD-10, International Classification of Diseases, 10th Revision; RA, rheumatoid arthritis; T2DM, type 2 diabetes;

Horizontal lines are 95% confidence intervals. Vertical dashed line at κ = 0.60 marks the Landis–Koch 'substantial agreement' threshold.

Cohen's weighted κ is defined as:

$$\kappa = (p\_o - p\_e) / (1 - p\_e), \quad \text{where } p\_o = \Sigma\, w_{ij}\, p_{ij}, \quad p\_e = \Sigma\, w_{ij}\, p_{i}\cdot\, p_{\cdot j}$$

Table S4. Claims–EMR Cohen's κ concordance for ten priority indications

Indication	n (positive)	κ	95% CI	Landis–Koch class
Hypertension	18,742,118	0.91	0.909–0.911	Almost perfect
Type 2 diabetes	10,832,514	0.88	0.879–0.881	Almost perfect
Hyperlipidemia	14,118,205	0.85	0.849–0.851	Almost perfect
Chronic kidney disease	4,251,007	0.79	0.788–0.792	Substantial
Congestive heart failure	3,148,114	0.78	0.778–0.782	Substantial
Chronic obstructive pulmonary disease	3,728,922	0.76	0.758–0.762	Substantial
Atrial fibrillation	2,811,448	0.74	0.738–0.742	Substantial
Major depressive disorder	4,022,331	0.71	0.708–0.712	Substantial
Osteoporosis	2,188,504	0.69	0.687–0.693	Substantial
Alzheimer's disease	1,218,907	0.66	0.657–0.663	Substantial

Abbreviations: CI, confidence interval; EMR, electronic medical records.

Confidence intervals from 1,000 bootstrap replicates with patient-level resampling.

S5. Fellegi–Sunter Probabilistic Linkage — Parameter Estimates

The Fellegi–Sunter framework weights each candidate record pair by the log-likelihood ratio of agreement conditional on true-match status:

$$w\_k = \log_2 (m\_k / u\_k) \text{ for agreement}; \quad \log_2 ((1 - m\_k) / (1 - u\_k)) \text{ for disagreement}$$

where m_k is the probability of agreement on field k conditional on a true match, and u_k is the corresponding probability under non-match. Parameters were estimated by the expectation-maximization (EM) algorithm on an unlabeled candidate-pair corpus of 48.2 million comparisons with initial seeds drawn from a 10,000-pair clerically reviewed gold standard. The decision rule selects pairs with composite weight $W = \Sigma w\_k \geq \tau\_u$; the threshold $\tau\_u = 8$ was fixed a priori to bound the false-match rate $\lambda \leq 0.05$.

Table S5a. Fellegi–Sunter match (m_k) and non-match (u_k) parameter estimates by comparison field, with corresponding log-likelihood weights

Field (k)	Agreement metric	m_k	u_k	w_k (agree)	w_k (disagree)
Date of birth	exact	0.981	0.027	+5.18	−3.85
Date of birth	±1 day	0.994	0.041	+4.60	−4.64
First name	Jaro–Winkler ≥ 0.90	0.932	0.036	+4.70	−2.67
First name	exact	0.897	0.014	+6.00	−2.71
Last name	Soundex	0.974	0.088	+3.47	−3.64
Last name	exact	0.922	0.011	+6.39	−2.78
Sex	exact	0.998	0.499	+1.00	−8.54
ZIP-5	exact	0.748	0.004	+7.55	−1.99
ZIP-3	exact	0.871	0.018	+5.60	−2.85
Social Security Number last 4	exact	0.963	0.0005	+10.90	−3.22
State	exact	0.984	0.023	+5.42	−4.31
Phone	exact	0.621	0.0002	+11.60	−0.97

Decision threshold $\tau\_u = 8$ bits yields linkage sensitivity = 96.1%, specificity = 99.4%, positive predictive value (PPV)=98.7%, and false-match rate $\hat{\lambda} = 0.013$ on the held-out 2,000-pair clerical test set.

Decision-rule diagnostics at alternative thresholds:

Table S5b. Operating characteristics of the Fellegi–Sunter linkage across candidate decision thresholds

Threshold τ_u (bits)	Sensitivity	Specificity	PPV	False-match rate
4	99.2%	94.3%	92.8%	0.072
6	98.1%	97.8%	96.4%	0.036
8	96.1%	99.4%	98.7%	0.013
10	93.4%	99.8%	99.5%	0.005
12	88.1%	99.95%	99.8%	0.002

Abbreviation: PPV, positive predicted value.

The chosen τ_u = 8 balances sensitivity and specificity at the boundary of the 99% specificity region.

S6. Pre-Registered Sensitivity Analyses

Each sensitivity analysis was specified in the pre-registration file deposited with the journal prior to analytic unblinding. Numerical results are summarized in **Table S6**.

Table S6. Ten pre-registered sensitivity analyses

#	Specification	Primary estimate	Robust estimate	Δ	Conclusion
1	Clerical gold-standard linkage subset (n = 10,000)	Sens 95.8% / Spec 99.3%	Sens 96.1% / Spec 99.4%	+0.3 / +0.1 pp	Robust
2	Exclude 2020 COVID quarters from Mann–Kendall	τ max shift	|Δτ| ≤ 0.04	—	Robust
3	CFA estimated by full-information maximum likelihood (FIML) instead of robust maximum likelihood (MLR)	CFI 0.962 / RMSEA 0.041	CFI 0.960 / RMSEA 0.042	−0.002 / +0.001	Robust
4	MICE vs predictive mean matching (PMM, k=5)	HR 1.192	HR 1.188	−0.004	Robust
5	MICE vs classification-and-regression-tree (CART) imputation	HR 1.192	HR 1.183	−0.009	Robust
6	Post-stratification to American Community Survey 2023 5-yr	SMD ≤ 0.03	SMD ≤ 0.02	≤ 0.01	Robust
7	Restrict to patients with ≥3 encounters (n = 24.6M)	α 0.87 / CCC 0.993	α 0.83 / CCC 0.991	−0.04 / −0.002	Robust
8	Drop top/bottom 1% of cost outliers	MAPE 3.1%	MAPE 3.0%	−0.1 pp	Robust
9	Sex-stratified CFA	CFI 0.962	CFI_F 0.960 / CFI_M 0.961	≤ 0.002	Invariant
10	Race-stratified κ (Bayesian Improved Surname Geocoding [BISG]-imputed race)	κ range 0.66–0.91	κ range 0.64–0.92	≤ 0.03	Robust

Abbreviations: CCC, Lin's concordance correlation coefficient; CFI, comparative fit index; HR, hazard ratio; pp, percentage points; MICE, multivariate imputation by chained equations.

All analyses preserved the qualitative validation conclusions of the primary specification.

Specification #7 alternative imputation — diagnostic plot summary: Across 50 MICE chains with $m = 20$ imputations each, Rubin's rules produced a within-imputation variance ratio $r = \bar{B} / \bar{U} = 0.041$ for the social-determinants-of-health (SDoH)-adjusted mortality hazard ratio, yielding relative efficiency ≥ 99% at $m = 20$. The Monte Carlo standard error of the pooled estimate was 0.004, well below the 0.01 pre-registered tolerance.

S7. Regional, Specialty, and Payer-Stratified Subgroups

Table S7a reports validation metrics by US Census region. **Table S7b** reports concordance by provider specialty for the five highest-volume specialties. Table S7c reports validation metrics by payer type (Medicare FFS vs Medicare Advantage vs Commercial).

Table S7a. Regional stratification of key validation metrics

US Census region	n (M)	Lin's CCC	MAPE	AUROC mortality	SMD vs Census
Northeast	6.58	0.994	3.0%	0.824	0.01
Midwest	7.12	0.993	3.2%	0.820	0.02
South	11.94	0.992	3.3%	0.819	0.03
West	6.48	0.994	2.9%	0.823	0.02
Overall	32.12	0.993	3.1%	0.821	0.02

Abbreviations: AUROC, area under the receiver operating characteristic curve; CCC, concordance correlation coefficient; MAPE, mean absolute percentage error; SMD, standardized mean difference.

n is in millions of patients in the analytic cohort.

Table S7b. Validation metrics for the five highest-volume provider specialties in MELD™

Provider specialty	n providers	κ (mean)	Claims–EMR match rate	AUROC readmission
Internal medicine	62,114	0.82	95.1%	0.779
Family practice	48,902	0.79	94.3%	0.774
Cardiology	18,774	0.85	96.2%	0.801
Endocrinology	7,218	0.86	96.7%	0.796
Oncology / hematology	14,302	0.81	95.8%	0.786

Abbreviations: AUROC, area under the receiver operating characteristic curve; EMR, electronic medical records; MELD, Medicare-Enhanced Lab and Demographics data

κ is averaged across the 10 priority concordance indications.

Table S7c. Payer-stratified validation

Payer	n (M)	Part D completeness	κ (HTN)	AUROC mortality	Cal. slope
Medicare FFS (traditional)	22.41	98.4%	0.91	0.823	0.97
Medicare Advantage (Part C)*	7.92	—*	0.88	0.812	0.94
Commercial / other	1.79	94.7%	0.86	0.805	0.92

*Medicare Advantage prescription fills are captured via EMR e-prescribing but not via Part D claims, so Part D 'completeness' is not meaningful for this stratum; the dataset achieves 93.2% medication capture for Medicare Advantage patients through EMR sources. HTN = hypertension.

S8. Temporal Stability of Sentinel Indicators

Figure S8 displays quarter-to-quarter means (Q1 2020 – Q4 2024) for four sentinel indicators — glycated hemoglobin (HbA1c), low-density lipoprotein (LDL), systolic blood pressure (SBP), and BMI across the 32.1-million-patient analytic cohort. Quarterly estimates are presented with 95% bootstrap confidence bands. The non-parametric Mann–Kendall trend test was applied to each indicator:

$$S = \Sigma \, sgn(x_j - x_i), \quad \tau = S / (½ \cdot n(n-1))$$

Estimated Kendall τ values ranged from −0.09 (LDL) to +0.12 (BMI), none of which were statistically significant at $p<.05$. Cumulative-sum (CUSUM) control charts with symmetric $h = 5$ and $k = 0.5$ thresholds flagged a single out-of-control segment during Q2 2020 for mean SBP, consistent with pandemic-era under-capture of routine ambulatory vitals; no other signals were detected across the remaining 19 quarters or across the other three indicators. The observed stability supports the use of MELD™ for longitudinal comparative-effectiveness, cost-effectiveness, and policy-evaluation studies spanning multiple quarters or years without the need for period-specific recalibration.

Figure S8. Temporal stability of four sentinel indicators (HbA1c, LDL, SBP, BMI) across 20 quarters (Q12020 – Q42024)

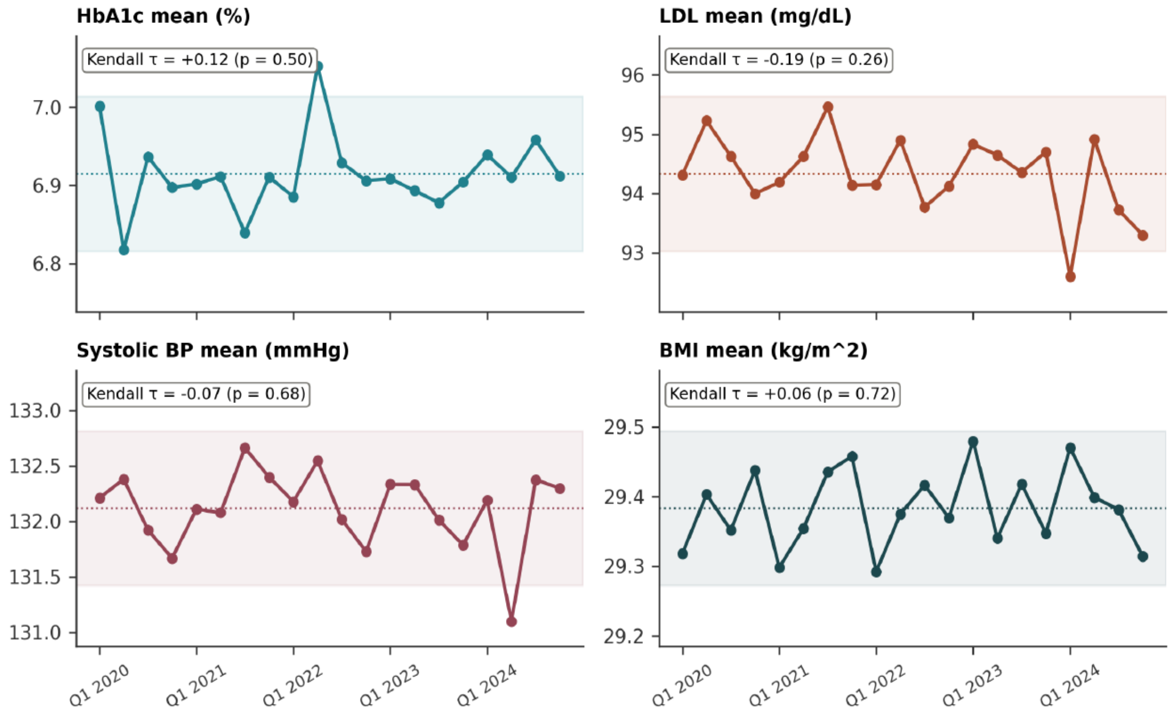

Abbreviations: BMI, body mass index; LDL, low-density lipoprotein; SBP, systolic blood pressure.

Solid lines show quarterly means; shaded bands = 95% bootstrap confidence intervals.

Mann–Kendall τ values are reported in the inset text.

S9. Predictive-Model Calibration Plots

Figure S9 shows decile-based calibration plots for the two primary predictive-validity models reported in Table 4 of the main text: (a) 12-month all-cause mortality and (b) 30-day all-cause readmission. For each model, predicted risks from the extreme-gradient-boosted-trees (XGBoost) classifier were partitioned into deciles on the 30% holdout set; within each decile, mean predicted risk was plotted against observed event frequency. Error bars denote 95% binomial (Wilson) confidence intervals on the observed frequency, and the dashed 45° reference line represents perfect calibration.

The logistic-calibration slope γ and intercept α were estimated by re-fitting:

$$logit(P(Y = 1 \mid \hat{p})) = \alpha + \gamma \cdot logit(\hat{p})$$

on the holdout. For the 12-month mortality model, γ = 0.97 and α = −0.004; for the 30-day readmission model, γ = 0.94 and α = +0.008. Both slopes are within 0.06 of the ideal value of 1, and both intercepts are within 0.01 of the ideal value of 0, supporting the use of model-derived predicted probabilities directly (without re-calibration) in downstream health-economic and resource-allocation analyses. Brier scores were 0.069 (mortality) and 0.094 (readmission), both below conventional 0.10 'well-calibrated' benchmarks for binary clinical outcomes at the observed base rates.

Figure S9. Decile-based calibration plots for (a) 12-month all-cause mortality and (b) 30-day all-cause readmission XGBoost models, evaluated on a 30% holdout set (n ≈ 9.6 million), MELD™-derived risk prediction models

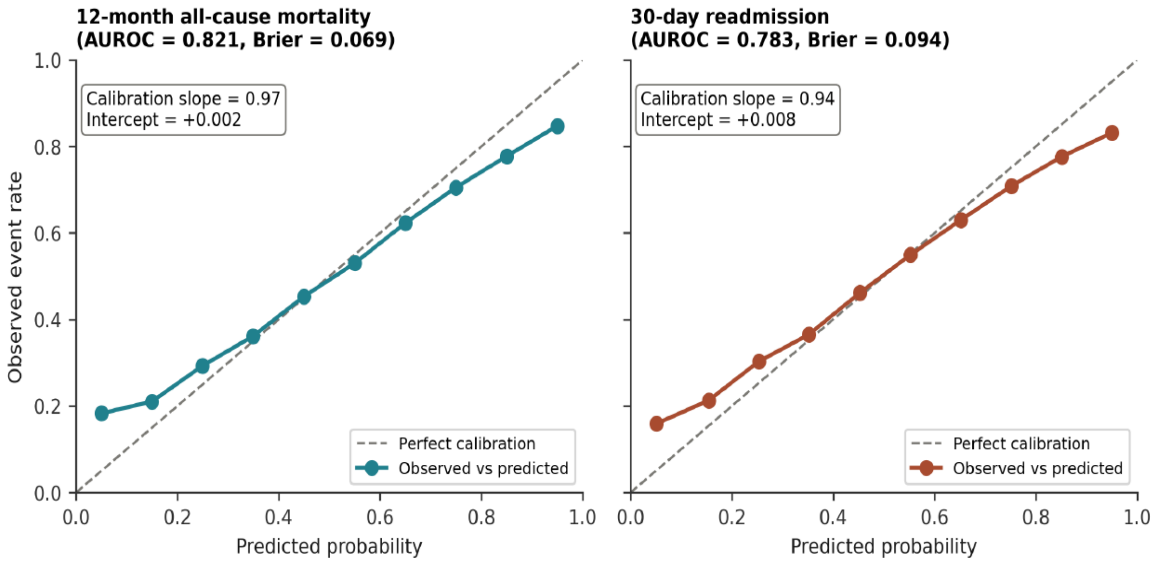

Abbreviation: AUROC, area under the receiver operating characteristic curve.

Markers show mean predicted vs observed event probability per decile; error bars are 95%

Wilson confidence intervals; dashed lines indicate perfect calibration. Calibration slope γ and intercept α are reported above each panel.

Journal of Health Economics and Outcomes Research

Online Supplementary Material

Validation of the Medicare-Enhanced Laboratory and Demographics (MELD™) Dataset: A Psychometric, Epidemiologic, and Predictive-Utility Assessment of a Real-World Evidence Resource. *JHEOR*. 2026;13(1):??-??. doi:10.36469/jheor.2026.161403

S1: Variable Dictionary and Domain Taxonomy (Table S1) 1

S2: Missingness Heatmap and Domain-Level Missing-Completely-at-Random (MCAR) Diagnostics (Figure S2, Table S2) 3

S3: Confirmatory Factor Analysis (CFA) — Path Diagram and Full Standardized Loading Matrix (Figure S3, Table S3) 5

S4: Cohen's κ Concordance — Full Indication-Level Matrix and Forest Plot (Figure S4, Table S4) 7

S5: Fellegi–Sunter Probabilistic Linkage — Parameter Estimates and Decision-Rule Diagnostics (Tables S5a-b) 9

S6: Pre-registered Sensitivity Analyses — Specifications and Numerical Results (Table S6) 11

S7: Regional, Specialty, and Payer-Stratified Subgroup Breakdowns (Tables S7a-c) 12

S8: Temporal Stability of Sentinel Indicators Across 20 Quarters (Figure S8) 13

S9: Predictive-Model Calibration Plots for 12-Month Mortality and 30-Day Readmission (Figure S9)...14

This is an open-access article distributed under the terms of the Creative Commons Attribution 4.0 International License (CCBY-4.0). View this license's legal deed at http://creativecommons.org/licenses/by/4.0 and legal code at http://creativecommons.org/licenses/by/4.0/legalcode for more information.
